# Determination of the Thermodynamic Parameters of the Pyrolysis Process of Post-Consumption Thermoplastics by Non-Isothermal Thermogravimetric Analysis

**DOI:** 10.3390/polym13244379

**Published:** 2021-12-14

**Authors:** Paul Palmay, Cesar Puente, Diego Barzallo, Joan Carles Bruno

**Affiliations:** 1 Facultad de Ciencias, Escuela Superior Politécnica de Chimborazo ESPOCH, Panamericana Sur Km 1 1/2, Riobamba 060155, Ecuador; cesar.puente@espoch.edu.ec; 2 Department of Mechanical Engineering, Universitat Rovira i Virgili, Avda. Paisos Catalans, 26, 43007 Tarragona, Spain; juancarlos.bruno@urv.cat; 3 Facultad Ciencias e Ingeniería, Universidad Estatal de Milagro, Milagro 091050, Ecuador; dbarzallog@unemi.edu.ec

**Keywords:** thermodynamic parameters, thermoplastics, degradation temperature, kinetic parameters

## Abstract

Currently, the pyrolysis process is an important technology for the final treatment of plastic waste worldwide. For this reason, knowing in detail the chemical process and the thermodynamics that accompany cracking reactions is of utmost importance. The present study aims to determine the thermodynamic parameters of the degradation process of conventional thermoplastics (polystyrene (PS), polyethylene terephthalate (PET), high-density polyethylene (HDPE), polypropylene (PP) and polyvinyl chloride (PVC)) from the study of their chemical kinetics by thermogravimetric analysis (TG). Non-isothermal thermogravimetry was performed at three heating rates from room temperature to 550 °C with an inert nitrogen atmosphere with a flow of 20 mL min^−1^. Once the TG data is obtained, an analysis is carried out with the isoconversional models of Friedman (FR), Kissinger–Akahira–Sunose (KAS), and Flynn–Wall–Ozawa (FWO) in order to determine the one that best fits the experimental data, and with this, the calculation of the activation energy and the pre-exponential factor is performed. The validation of the model was carried out using the correlation factor, determining that the KAS model is the one that best adjusts for the post-consumer thermoplastic degradation process at the three heating rates. With the use of the kinetic parameters, the variation of the Gibbs free energy is determined in each of the cases, where it is necessary that for structures containing aromatic groups a lower energy is presented, which implies a relative ease of degradation compared to the linear structures.

## 1. Introduction

The growing demand and dependence on plastics due to their great applicability in various sectors has caused an increase in the generation of plastic waste that will probably grow as a result of the problem of eliminating plastics [1]. Thus, the United Nations Development Program (UNDP) establishes a considerable reduction in waste generation through prevention, reduction, recycling, and reuse activities [2]. In this context, the trend is to change the current paradigm from a linear economy to a circular economy for the reuse and exploitation of plastic waste, achieving a better balance and harmony between the economy, the environment, and society [3].

Thus, the two main sources of plastic waste come from industries in the form of packaging waste and urban solid waste, whose presence in the environment is found in a greater proportion in the form of thermoplastics [4]. Promoting the use of sustainable and efficient alternatives that allow solving the environmental pollution problems that exist in incineration and dumping is the most important challenge. Although there are several alternatives for the management of plastic waste, the contributions dedicated to the recovery of energy through pyrolysis have grown notably in recent years due to their many operational and environmental advantages [5]. The pyrolysis process allows the anaerobic thermochemical decomposition of plastic waste at high temperature with or without the presence of a catalyst to obtain biofuels, which are characterized by their high calorific values that coincide with commercial fuel and involve a minimization in the carbon footprint of plastic products by reducing carbon dioxide and monoxide emissions [6].

Currently, the production of fuels has been obtained from individual thermoplastic waste such as polypropylene (PP) [7,8], polystyrene (PS) [9], polyethylene terephthalate (PET) [10], polyethylene (PE) [10,11], polyvinyl chloride (PVC) [12], and mixed plastic waste [13,14], with characteristics similar to fossil fuels and whose performance depends mainly on the raw material fed and the rate of heating at which it is developed. In this context, it is necessary to understand the reaction kinetics of the thermal degradation of plastics to determine the main kinetic parameters that govern the reaction in the pyrolytic reactor and thus predict the stability of plastic waste [15]. This thermal degradation process is commonly evaluated by thermogravimetric analysis (TGA) under non-isothermal (dynamic) conditions, using different heating rates to establish the global or macroscopic kinetics of the process. With the data obtained from the TGA, the determination of the kinetic triplet is sought: activation energy, reaction order, and pre-exponential factor (frequency factor), which describe the pyrolysis process [16].

However, the mechanism of thermal degradation of plastics is complex in nature, and the International Confederation of Thermal Analysis Calorimetry (ICTAC) recommends isoconversional methods (differential or integral method) to evaluate the degradation kinetics of plastic waste by calculating the activation energy as the reaction proceeds with the experimental data obtained from the TGA considering a general degradation reaction [17].

In this sense, different isoconversional methods have been used to evaluate the thermal decomposition of PP [18], HDPE [19], PVC [20,21], PS, and PP [22]. The kinetics of the pyrolysis process and the corresponding isoconversional models have important significance in the conversion processes of post-consumer thermoplastics, which are studied as general reactions for ease of study [23]. Several authors have presented studies for the determination of the chemical kinetics of the degradation process of thermoplastics using models such as Friedman, Kissinger–Akahira–Sunose (KAS), Flynn–Wall–Ozawa (FWO) at low heating rates using models of reaction in solid state, presenting activation energy results for each model, respectively, of the HDPE polyolefins of 247,238,243 kJ × mol^−1^ and PP of 188,179,183 kJ × mol^−1^ [24]. Another study [25] shows results of 238 kJ × mol^−1^ for PS and 238 kJ × mol^−1^ for PVC for the first degradation curve and 243 kJ × mol^−1^ for the second curve, using isoconversional methods which show important results in the study and optimization of the thermal degradation of the parameters and the structure of the products resulting from the pyrolysis process, such as coal, gas, and fuel.

In this context, in the plastics pyrolysis process, the energy associated with its various stages that occurs in different plastics under different conditions can be quantified by energy changes throughout the pyrolysis process. Therefore, the objective of the present study is to determine the thermodynamic parameters calculated from the kinetic parameters of the reaction, such as the energy change during the chemical reaction, called the Gibbs free energy change (ΔG), consisting of two terms, the enthalpy or heat of reaction (ΔH) and the temperature-dependent entropy (TΔS) [26].

## 2. Materials and Methods

### 2.1. Materials

The different post-consumer plastic waste used in this work was acquired from the existing garbage dump in the city of Riobamba, Ecuador. These residues were collected in different periods of time for ten days for three consecutive months, with an average total mass of 30 kg.

The samples were classified according to their origin, according to the corresponding thermoplastic family. These thermoplastics were crushed to a particle size between 3 and 4 mm, with a subsequent washing with a 10% sodium hydroxide solution (Scharlau) in tanks with jets of water under pressure and agitation, in order to eliminate waste residues—dust, labels, and glue. From the dry plastic waste, a sample mass of around 500 g of each type of plastic was obtained, which were classified into fractions of polypropylene (PP), compact polystyrene (PS), polyethylene terephthalate (PET), high-density polyethylene (HDPE), and polyvinylchloride (PVC).

### 2.2. Physicochemical Characterization of the Waste

The classified plastic waste has been characterized by Fourier Transform Infrared Spectroscopy (FTIR) using a JASCO FT/IR-4100 spectrometer. The method used has been executed with the Spectra Analysis program, which performs the data acquisition and treatment, and provides a numerical value based on the height or area of the peak in a working scan range of 4000 to 550 cm^−1^.

### 2.3. Thermogravimetry of Thermoplastics

The study of the degradation kinetics of the different thermoplastics has been carried out by thermogravimetric analysis (TGA) to obtain the data on the loss of mass of the polymer with respect to time and temperature. The data obtained from the TGA has subsequently made it possible to calculate the kinetic triplet of the process: activation energy, pre-exponential factor, and reaction order. Thus, the temperature profiles of each residue have been obtained by using a 1 STAR System thermogravimetric analyzer (METTLER TOLEDO, Columbus, OH, USA) at three heating speeds (5, 10, 15 C × min^−1^), heating from room temperature to 800 °C, in an inert atmosphere, with a constant nitrogen injection flow of 20 mL × min^−1^ with maximum samples of 50 mg. The heating rates were chosen considering the slow pyrolysis conditions, which can be replicated in small reactors on a laboratory scale for the purpose of subsequent validations. Finally, the graphs of the remaining weight and the rate of weight loss as a function of temperature were obtained for each heating rate of each of the thermoplastics.

### 2.4. TGA Data Processing

The data obtained from the thermogram was reported as the variation of the mass with respect to time during heating; however, isoconversional methods use conversion values (mass loss fraction), which are converted by the following expression:

(1) $$\alpha =\frac{w_{i}-w_{\;}}{w_{i}-w_{f}}$$

where *α* is the conversion, $m_{i}$ is the initial mass, $m_{\;}$ is the mass at a given degradation time, and $m_{f}$ is the final or residual mass, which will vary in the range from 0 to 1. The variation of the conversion with respect to the time $\frac{d\alpha }{dt}$ is measured experimentally with the DTG, which is a function of the heating rate *β*, which is related by the expression:

(2) $$\frac{d\alpha }{dt}=\left(\frac{dT}{dt}\right)\left(\frac{d\alpha }{dT}\right)=\beta \left(\frac{d\alpha }{dT}\right)$$

Regarding the reaction rate (chemical kinetics) of the thermal degradation of plastic waste, it is established that the conversion rate is proportional to the concentration of the reactant and can be expressed as a function of temperature and conversion [27]:

(3) $$\frac{d\alpha }{dt}=\beta \frac{d\alpha }{dT}=k\left(T\right)f\left(\alpha \right)$$

where *k*(*T*) is the kinetic constant as a function of temperature, which is based on the Arrhenius equation, and f(*α*) is the reaction model as a function of conversion, which determines the reaction mechanism of thermal degradation.

(4) $$k\left(T\right)=A\;e^{\left(-\frac{E}{RT}\right)}$$

where *A* is the pre-exponential factor (s^−1^), *E* is the activation energy (kJ × mol^−1^), *R* is the gas constant (0.008314 kJ × mol^−1^ × K^−1^).

Although there are different reaction models with a characteristic kinetic curve, the most common example of a reaction model is defined here, where the conversion function depends on the reaction order:

(5) $$f\left(\alpha \right)={\left(1-\alpha \right)}^{n}$$

Considering the TG data set with the same value of *α* at different temperatures, the function f(*α*) becomes constant, and the parameters of the pre-exponential factor and the activation energy of the degradation processes become independent of the form from *f* [28]. According to this, Equation (3) would be expressed as:

(6) $$\ln\frac{d\alpha }{dt}=\ln\;\left(k\left(T\right)\right)+\ln{\left(1-\alpha \right)}^{n}$$

where for each temperature, the graph $\ln\frac{d\alpha }{dt}$ is made against ln(1−α) that provides a slope, which involves the apparent activation energy and the intercept to the value of the pre-exponential factor.

#### 2.4.1. Kinetic Models of Thermoplastics

From the information provided on the TG analysis data, in order to arrive at Equation (6), different isoconversional kinetic models (differential or integral) have been proposed that adjust to the experimental data to validate which of these fits the most for the case of sampled plastics [29].

#### 2.4.2. Kinetic Model 1: Friedman Method (FR)

This is a differential isoconversional method proposed by Friedman, which is directly based on Equation (6) [25], through the following expression:

(7) $$\ln\left(\frac{d\alpha }{dt}\right)=\ln\left(\beta \frac{d\alpha }{dT}\right)=\ln\left(A\right)-\frac{E}{RT}+\ln\left({\left(1-\alpha \right)}^{n}\right)$$

#### 2.4.3. Kinetic Model 2: Kissinger–Akahira–Sunose (KAS) Method

It is an isoconversional integral method, obtained by adjusting the integral of Equation (6):

(8) $$\frac{d\alpha }{f\left(\alpha \right)}=\frac{A}{\beta }\;e^{\left(-\frac{E}{RT}\right)}\;d\left(T\right)$$

where the first term is expressed in a new function:

(9) $$\frac{d\alpha }{f\left(\alpha \right)}=g\left(\alpha \right)$$

Which is integrated with the initial conditions of *α* = 0 in *T* = *T*_0_, and assuming that *A*, f(*α*), and *E* are independent of *T*, while *A* and *E* are independent of *α*, we have:

(10) g(α)=AEβT[e(−ERT)ERT−∫−∞ERTe(−ERT)ERTd(T)]

The expression that incorporates the new function g(*α*) can be adjusted in the relation:

(11) P(ERT)=e(−ERT)ERT−∫−∞ERTe(−ERT)ERTd(T)

This equation that can be solved based on approximations such as Coats–Redfern, where *P*$\left(\frac{E}{RT}\right)$, is replaced and linearized by applying logarithm, and the reaction model is included, obtaining the following expression:

(12) $$\ln\frac{\left({\left(1-\alpha \right)}^{n}\right)}{T_{m}{}^{2}}=\ln\frac{AR}{E}-\ln\beta -\frac{E}{RT}$$

For which $\;\frac{\left({\left(1-\alpha \right)}^{n}\right)}{T_{m}{}^{2}}$ versus 1/*T*; the activation energy (*E*) is obtained from the slope, and the pre-exponential factor (*A*) is obtained from the point of the order.

#### 2.4.4. Kinetic Model 3: Flynn–Wall–Ozawa (FWO) Method

This model is very similar to the previous one, which differs in the resolution of the integral of Equation (10), in which the Doyle approximation [30] is used, leaving for $\ln P\left(\frac{E}{RT}\right)$:

(13) $$\ln P\left(\frac{E}{RT}\right)=-5.331-1.052\frac{E}{RT}\;$$

Relating Equations (11) and (13), and applying the reaction model for g(*α*), we have:

(14) $$\ln\left({\left(1-\alpha \right)}^{n}\right)=\ln\frac{AR}{E}-\ln\beta -5.331-1.052\frac{E}{RT}\;$$

It is graphed $\left({\left(1-\alpha \right)}^{n}\right)$ vs. 1/*T*; the activation energy (*E*) is obtained from the slope, and the pre-exponential factor (*A*) is obtained from the point of the order.

#### 2.4.5. Reaction Model

The use of isoconversional models without establishing the reaction model or with the blind assumption of a model leads to the calculation of an activation energy distribution that does not contemplate a reaction model that follows the process at different heating rates. By means of the Criado method, which compares the experimental results of TGA with the reaction models, the model is determined for each case study as shown in Table 1 [23,30].

### 2.5. Validation and Tuning of the Models

To determine the kinetic parameters of the material, we worked with a range of degrees of advance x, in such a way that the linear adjustment is conducive to carrying out the analysis of the TGA data. The linear area of *α* was determined by representing d*α*/dt or *β* d*α*/d*T* as a function of *α*. If you want to obtain a good linear fit, it is recommended to work with a range of *α* lower than the maximum point (this generally ranges from 0.4–0.6). It should be mentioned that this range coincides with the beginning of the degradation reaction of several thermoplastics, which is why it is very useful to determine the kinetic parameters of the material [31].

The adjustment of the models has been carried out by means of the optimization by the method of least squares, while the validation and comparison between the three proposed models are defined by determining the correlation coefficient between the experimental data and the theoretical data.

#### Thermodynamic Parameters

In the thermal pyrolysis process of the different thermoplastics used, the thermodynamic parameters of the material provide information on the feasibility or spontaneity of the process itself at the different operating conditions that may occur.

Thus, the calculation of the enthalpy change, which represents the total energy consumed by the material for its conversion into the different fractions or various products, such as fuel, gas, and coal [32], would be determined with the following equation that is based on the calculation of the activation energy related to the macroscopic decomposition kinetics:

(15) $$\Delta H=E_{a}-RT\;$$

While the Gibbs free energy and the entropy of the process is calculated by:

(16) $$\Delta G=E_{a}+RT_{m}*\ln\left(\frac{K_{B}*T_{m}}{h*A}\right)$$

where Δ*G* is the Gibbs free energy, and *K_B_* and *h* are the Boltzmann and the Planck constant, respectively.

Finally, the entropy (ΔS) indicates the degree of disorder of the material, which is expressed as:

(17) $$\Delta S=\frac{\Delta H-\Delta G}{T_{m}}\;$$

## 3. Results

### 3.1. Characterization of Samples

FTIR has been used for the chemical characterization of different plastic wastes. A summary of IR linkages for functional group analysis is described in Table 2.

**Figure 1:**
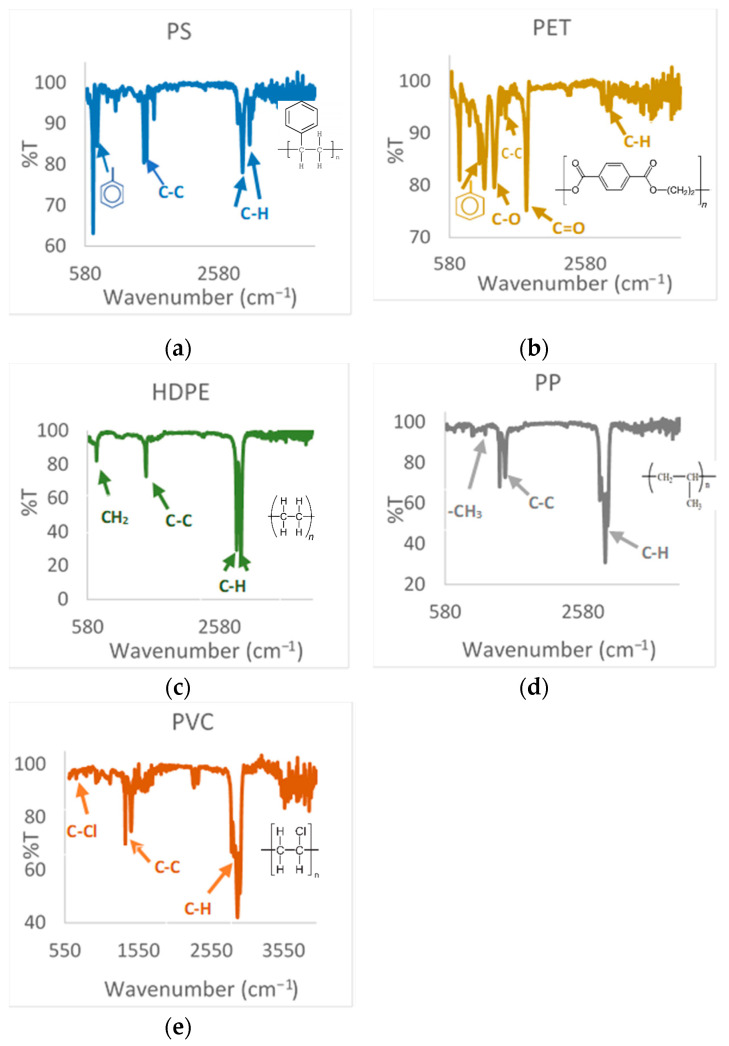
FTIR spectra curves of: (**a**) PS, (**b**) PET, (**c**) HDPE, (**d**) PP, (**e**) PVC.

In Figure 1, the FTIR spectra curves of PS, PET, HDPE, PP, and PVC are shown. In the PS spectrum, three groups of bands can be seen corresponding to the multiple tension movements of the CH bonds at 2700–3000 cm^−1^, CC at 1400–1600 cm^−1^ of the aromatic ring, and a bending movement of -CH_2_ and aromatic ring tensions between 700–800 cm^−1^, according to what is stated in the bibliography [30]. In the PET spectrum, an intense band corresponding to the tension of the C=O bond at 1700 cm^−1^ and tension movements between 1000 and 1100 cm^−1^ are visualized by tensions of the aromatic ring bonds; however, the bands of CH bond tensions at 2800–2900 cm^−1^ are weak, although they can be seen in relation to the results presented by a similar study [33]. In the HDPE spectrum, three groups of bands are clearly observed, corresponding to the tension movements of the C-H bonds between 3000–2700 cm^−1^, C-C tension at 1465 cm^−1^, and a bending movement of CH_2_ at 717 cm^−1^. Chemically, HDPE is the same as LDPE; therefore, the absorption bands are the same, characteristic of this plastic [29]. In the PP spectrum, three groups of bands are clearly observed corresponding to tension movements of the CH bonds at 2900 cm^−1^, DC tension movements at 1350–1470 cm^−1^, and a bending movement of –CH_3_ between 1200 and 1000 cm^−1^. Lastly, the PVC spectrum presents some similarity with respect to the PET spectrum due to the position of the transmittance bands; however, less strong bands are observed between 1000 and 1100 cm^−1^ due to the effect of the presence of chlorine. A band of tension of the C-H bonds is also observed at 2850–2900 cm^−1^, and another of C-C tension at 1350–1470 cm^−1^.

Finally, it is important to note that in some spectra, a small band very close to 1470 cm^−1^ is observed, which is typical of azotriazoles, the base structure of additives that are added to single-use plastics or commercial plastics as UV absorbers [34,35]. These structures that are added in low percentages and that have a low molecular weight will not interfere in the reaction mechanism of the degradation process; however, due to the high temperatures and the generation of intermediates caused by the incision of the beta bond, it can generate final compounds in recoverable liquid products from pyrolysis.

### 3.2. TGA Data Processing

**Figure 2:**
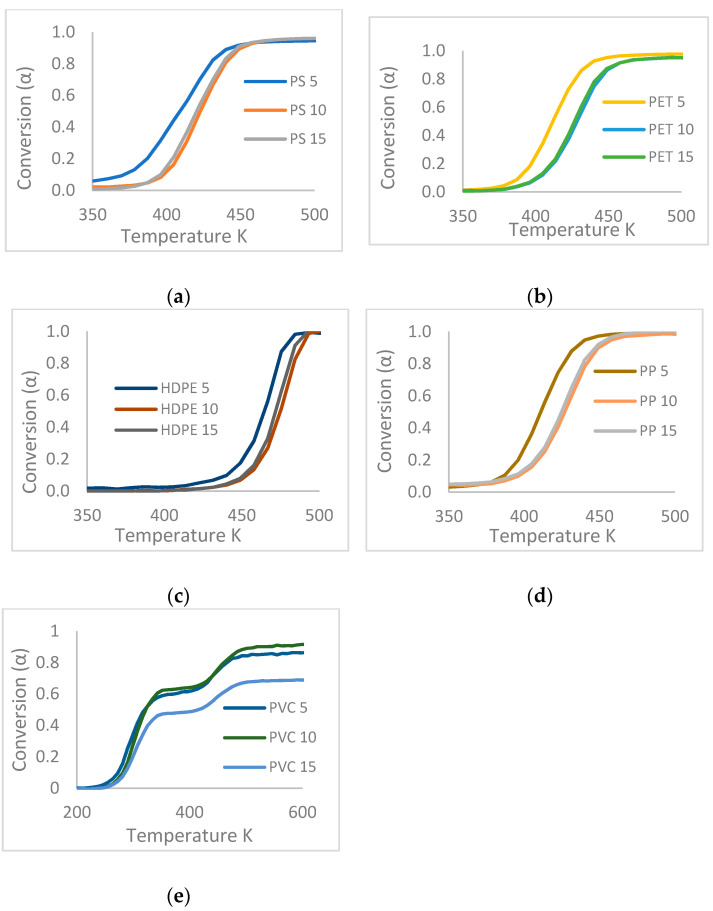
Thermal degradation profile for TGA of (**a**) PS, (**b**) PET, (**c**) HDPE, (**d**) PP, and (**e**) PVC at three heating speeds (5, 10, 15 K × min^−1^).

In Figure 2, the TGA data for PS, PET, HDPE, PP, and PVC are shown at different heating rates (5.10 and 15 K × min^−1^), where it is observed that for each plastic waste the slope of the curve of the loss of weight does not change with the variation of the heating rate; there is only a displacement towards the zone of lower temperature at a lower heating rate, due to the better diffusion of heat and mass that occurs at the time of the addition of heat over a longer period.

In addition, it is noted that there is the same shape of the curve for PS, PET, HDPE, and PP, which indicates that they have the same pyrolysis behaviors due to similar chemical bonds in their molecular structures with a degradation reaction in a single step due to its combination with all the reactions that are carried out, and this shows a general conversion of degradation [4,23]. In the case of the TG curve of PVC, it can be seen that the conversion takes place in two stages [28], a first between 50 and 60% weight loss that corresponds to a first reaction for the dehydrochlorination of the structure at a temperature between 270 and 350 °C with a first higher peak that can be explained by the stability of the CC bonds with respect to CX bonds, and a latter that will be broken faster and with higher priority for subsequent cracking and decomposition of the polymer when the PVC is pyrolyzed [27,35]. Additionally, the appearance of a small peak at a temperature higher than 650 °C can be observed, which is due to the energy consumed for the reorganization of the structures generated in the cracking process of the macromolecule.

**Figure 3:**
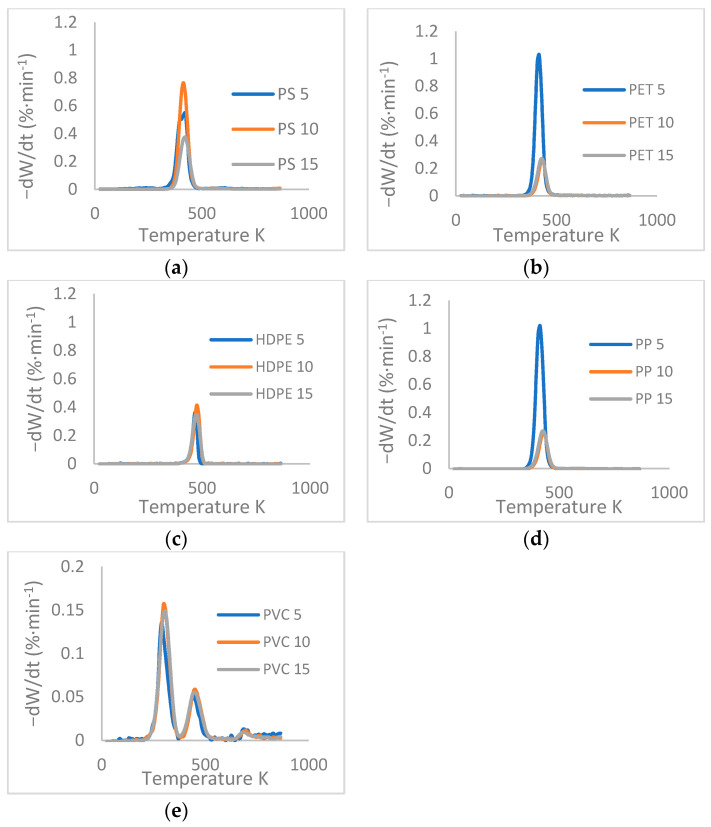
DTG curves of different plastics: (**a**) PS, (**b**) PET, (**c**) HDPE, (**d**) PP, (**e**) PVC at three heating speeds (5, 10, 15 K × min^−1^).

The foregoing is corroborated from the DTG curves of Figure 3, where it is confirmed that PS, PET, HDPE, and PP plastics have a very similar conversion slope with slight displacement to higher temperature zones, especially for HDPE, according to what is stated in the bibliography [36]. In addition, it is noted that there is a solitary peak in some DTG graphs, due to the general degradation that occurs in a continuous process. In reality, the thermal degradation of plastics proceeds as a random beta bond cleavage reaction that entails several steps, including in series and parallel reactions. Finally, as shown in the previous figure, it is observed that the thermal degradation process of PVC is carried out in two stages with respect to the other plastic wastes.

### 3.3. Kinetic Analysis

Table 3 presents the activation energy and the pre-exponential factor obtained by the three isoconversional models used at the different heating rates of 5, 10, and 15 K × min^−1^ of PS, PET, HDPE, PP, and PVC. All the proposed models present values with slight differences between them that are given by the mathematical approximations used. In this context, two sets of data obtained for PVC are presented, since it has been analyzed separately due to the two stages in which the pyrolysis process of this plastic takes place; PVC 1 refers to stage 1, and PVC 2 to stage 2.

**Figure 4:**
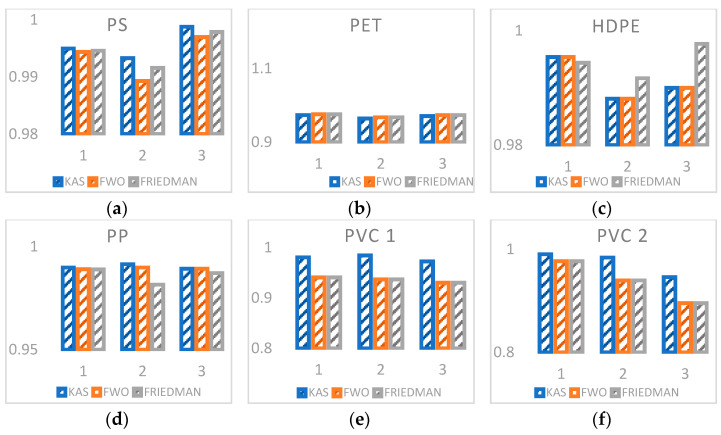
Comparison of the different isoconversional methods based on the linearity coefficient (R2) for the different heating rates of different plastics: (**a**) PS, (**b**) PET, (**c**) HDPE, (**d**) PP, (**e**) PVC 1, (**f**) PVC 2. Values 1, 2, 3 on the *x*-axis mean heating rates of 5, 10, 15 K × min^−1^, respectively.

In Figure 4, a comparison of the three models proposed for each of the plastics is shown based on the correlation coefficients of data obtained at different heating rates. In all cases, a great relationship is observed between the data of the proposed model and the experimental data (very close to one); however, despite the fact that Friedman’s model is a differential model, the distribution of the apparent activation energy to different conversions presents a greater deviation than the integral models [23]. In this context, it is observed that the KAS model presents a higher correlation value compared to the other two models, since the maximum degradation temperature of the process is integrated in the analysis of the reaction kinetics.

**Figure 5:**
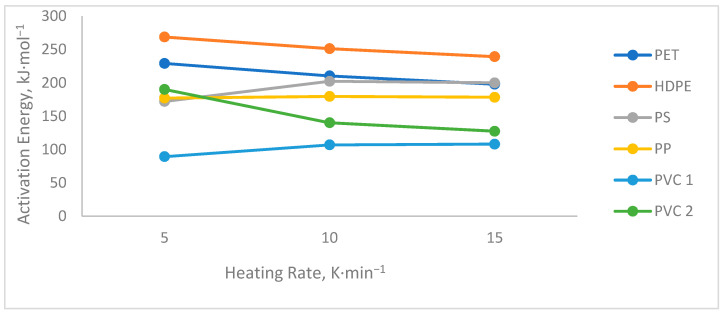
DTG curves of different plastics at three heating speeds (5, 10, 15 K × min^−1^).

The activation energy values obtained at different heating rates of the different plastics that differ due to their molecular structures are shown in Figure 5. In polyolefins, HDPE, PP, and PS thermal stability is affected by the type of carbocation that is generated with respect to the branching that the base structure presents. In PP and PS, there are weak links due to the presence of a tertiary carbon at the beginning of the degradation reaction at any rate of heating, which is why their general activation energy has a similar behavior. For HDPE, there is a higher activation energy profile caused by the energy consumption necessary to generate the breakdown of the CC bond (350 kJ × mol^−1^) after the random incision, which is carried out at temperatures greater than 400 °C [23,28]. This corroborates the proper use of the degradation kinetic models that for HDPE and PP were R2 and R3, respectively [27,37], since both R2 and R3 are geometric contraction models and assume that the rate of the degradation reaction begins at the surface and the rate is controlled by the progress of the resulting interface reaction towards the center. R2 and R3 differ in the shape of the particles; R2 is generally considered as a contraction cylinder or contraction area, and R3 represents a contraction sphere or contraction volume [38], thus generating a greater or lesser energy consumption for the generation of low-molecular-weight molecules due to its decomposition and depending to a great extent on the diffusion of heat and mass that occurs in the reactor, reliant on the characteristics of the reactor and the rate of heating.

The behavior of PS with PET is very similar due to the presence of the aromatic ring in its structures. The increase in activation energy in the case of PS at a heating rate of 10 K × min^−1^ is given by the passage to the degradation initiated by random cleavage more rapidly. Taking into account that the degradation of plastics actually involves the breaking of the bonds between the individual atoms that make up the polymer chain (CC~350 kJ × mol^−1^) and requires a higher activation energy, and the degradation occurs above 400 °C at low heating rates where heat diffusion is slower, degradation can easily begin due to thermally labile bonds (weak links such as branches and head-to-head links) inherent in the polymer chain [23].

As for PVC, it is a particular case within thermoplastics since it presents a degradation reaction that takes place in two stages—including a first stage of elimination of chlorine, with a more pronounced DTG peak and a lower activation energy consumption compared to the second. This second stage is the part of the degradation in which the decomposition into smaller hydrocarbons occurs, with a higher energy consumption at a higher temperature. This difference is less accentuated in a higher heating rate (15 K × min^−1^), since the addition of heat occurs more quickly, generating a partial elimination of chlorine, while the pyrolytic decomposition begins that can generate compounds with halogen radicals [30,36].

The breaking of the molecular bonds will depend on each polymer with respect to the temperature used. In this context, at a higher temperature, the heating rate will be higher, and this will affect the kinetic parameters, as shown in Figure 5, in the values obtained in the activation energy at different heating rates for each polymer. An increase in activation energy can be observed in some polymers (PS, PS, and PVC 1) when the heating rate has reached 15 K min^−1^. However, the activation energy is almost constant in each polymer when it reaches this heating rate, similar to the results reported by [39]. The reason for this constant distribution is that at high temperatures, the C-C bonds break more easily, generating more short-chain hydrocarbons [40] and aromatic hydrocarbons [41]. Furthermore, after reaching the maximum degradation temperature or close to it, only secondary reactions occur between the radicals present in equilibrium.

### 3.4. Thermodynamic Parameters

Table 4 shows the enthalpy consumption data, entropy changes, and Gibbs free energy for each of the plastics at the different heating rates with the data obtained from the KAS kinetic model, the model that presented the best fit.

In the case of PVC, the thermodynamic parameters have been determined for each peak of the process, presenting an enthalpy of the first peak of 85 kJ × kmol^−1^; a low energy consumption compared to other plastics, which was carried out without major setback at low temperatures (550 K) with a Gibbs free energy (162 kJ × kmol^−1^), which would be understood as a preparation (elimination of chlorine) to proceed to the formation of the activated complex; and the initiation of the degradation of the base of the polymeric structure.

Furthermore, since ΔH is the predominant parameter in ΔG, the type of reaction can be distinguished by the value of ΔH, because ΔG = ΔH − TS. In a reaction, if enough bond dissociation energies are known, then ΔH can be calculated. For a given plastic and given pyrolysis conditions, the value of ΔH depends on the composition of the final products obtained from the pyrolysis process [26]. For example, the bonds in PE are quasi-secondary C-C bonds with a bond dissociation energy of 355 kJ × mol^−1^. Straight-chain hydrocarbons are the predominant products generated from the PE pyrolysis process. Therefore, in the reactions, many C=C double bonds and primary C-C bonds have been produced at the ends of the chain; 1-alkene, n-alkane, and *α*, ω-dialkene are the main hydrocarbons produced. The bond dissociation energies of the primary C-C bond and the C=C double bond are 376 and 611 kJ × mol^−1^, respectively. Both types of product bonds formed require more energy than the energy released by breaking the secondary C-C bond in the reactant, 355 kJ × mol^−1^. Therefore, the PE pyrolysis process is an endothermic reaction that can be confirmed by findings from previous studies [42].

**Figure 6:**
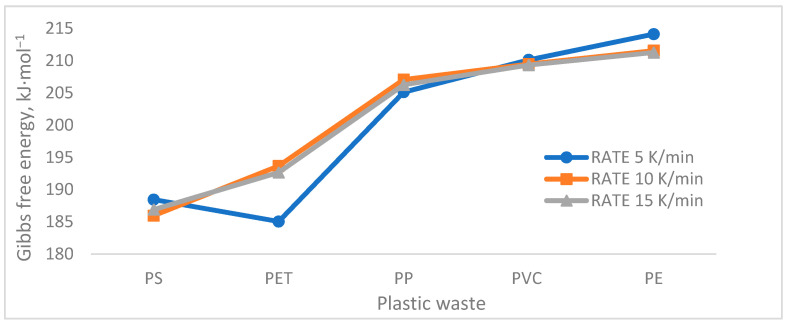
Gibbs free energy change.

In Figure 6, the Gibbs free energy is represented for the three heating rates of the thermoplastics studied, where the tendency in the cracking of the structures (PS and PET) can be noted, due to the presence of an unstable carbocation, which corresponds to the aliphatic chain in its structure [43], at the moment that the radical is generated by the controlled addition of temperature at any rate of heating compared to other plastics. Followed by PP and PVC, which present intermediate values for the tertiary carbocation generated, PE is the plastic that presents a higher Gibbs free energy. Although there is not a great difference between plastics, measuring the thermodynamic ease for the pyrolysis of these products to take place is established as an important indicator.

On the other hand, if thermal degradation is considered as a single-stage process, PS, PP, and PVC would degrade in the same way at any rate of heating; however, in the case of PET, there is a notable difference of about 8 kJ/mol at the lowest heating rate (5 K/min). This is because PET presents a greater difficulty in breaking its polymeric structure, due to the presence of COO- groups in its main chain, which requires more time and energy for the formation of the activated complex than at higher heating rates [44]. In the case of PE, it is easier at high heating rates, as moving to the area of higher energy would lead to greater bond breakage, as it is a linear structure [45].

## 4. Conclusions

Pyrolysis as a final treatment technique for plastics is a very important process today, which is why the study of the parameters of influence in said process is of utmost importance. In this sense, the activation energy was determined by thermogravimetric analysis. The pre-exponential factor was used for the main thermoplastics for mass consumption, since these are the waste with the highest weight in garbage dumps. The model that best adjusted for this type of recycled materials was the KAS model. From the kinetic data of the decomposition of PP, PS, HDPE, and PVC, the thermodynamic parameters were determined, with a focus on the analysis of Gibbs free energy as a factor of relevance to determine the “ease” of pyrolyzing plastic compounds. It was obtained that the structures that contain aromatic groups in their PS and PET structure have a lower Gibbs value compared to polymers that have linear structures. On the other hand, the results show that low heating rates (5 °C × min^−1^) present less “ease” of processing, while under a rate of between 10 and 15 °C × min^−1^, they do not present significant differences, though show a greater “ease” compared to 5 °C × min^−1^.

## Figures and Tables

**Table 1:** Reaction models of each plastic waste.

Plastic Waste	Reaction Model	Model Code	f(*α*)	g(*α*)
HDPE	Contracting cylinder: two-dimensional phase boundary reaction	R2	2(1 − *α*)^1/2^	1 − (1 − *α*)^1/2^
PP	Contracting cylinder: three-dimensional phase boundary reaction	R3	3(1 − *α*)^2/3^	1 − (1 − *α*)^1/3^
PS	Avrami–Erofeev: two-dimensional nucleation	A2	2(1 − *α*) × [−ln(1−*α*)]^1/2^	[−ln(1 − *α*)]^1/2^
PET	Power law	P2	2(*α*)^1/2^	*α* ^1/2^
PVC	Three-dimensional diffusion	D3	3/2(1 − *α*)^2/3^[1 − (1 − *α*)^1/3^]^−1^	[1 − (1 − *α*)^1/3^]^2^

**Table 2:** Assignments of IR bands to vibratory modes in the atomic group.

Assigned Wave Number/cm^−1^	Group	Vibrating Mode
800–600	-C-Cl	Stretching
909–670	Mononuclear aromatic hydrocarbons	C-H bending force of the plane
1000–650	AR-H=C-H	Deformation vibration
1000–675	-C=C-	Bending
1000–800	=C-H	Bending
1000–960; 940–900	R-CH=CH2	Bending
1300–1000	Mononuclear aromatic hydrocarbons	Bending in plane
1300–1000	C-O	Stretching
1380–1460	-CH3	C-H bending
1460	-CH2-	Scissor
1470–1350	-CH3	Bending
1500–1400	Mononuclear aromatic hydrocarbons	Skeletal vibrations
1680–1600	-C=C-	Stretching
1750–1715	C=O	Stretching
2400–2100	-C≡C-	Stretching
3000–2700	-C-H	C-H stretch
3100–2600	H-Cl	Asymmetric stretch
3100–3000	=C-H	Stretching
3300–3000	Mononuclear aromatic hydrocarbons	C-H stretch
3300–3270	C≡C	Stretching

**Table 3:** Kinetic parameters obtained by isoconversional methods of different plastic wastes.

Plastic Waste	Model	*β* (°C × min^−1^)	*Ea* (kJ × mol^−1^)	*A* (K^−1^)
PS	KAS	5	172.02	8.41 × 10^11^
		10	202.22	2.41 × 10^14^
		15	199.93	1.37 × 10^14^
	FWO	5	170.38	1.42 × 10^13^
		10	167.68	4.65 × 10^13^
		15	205.61	4.27 × 10^12^
	FRIEDMAN	5	174.64	1.34 × 10^12^
		10	168.26	4.78 × 10^13^
		15	210.19	4.31 × 10^12^
PET	KAS	5	229.04	3.08 × 10^16^
		10	210.33	2.52 × 10^14^
		15	197.87	3.51 × 10^13^
	FWO	5	229.07	3.10 × 10^16^
		10	210.36	2.53 × 10^14^
		15	197.44	3.26 × 10^13^
	FRIEDMAN	5	229.05	3.08 × 10^16^
		10	210.35	2.52 × 10^14^
		15	197.87	3.51 × 10^13^
HDPE	KAS	5	268.62	1.09 × 10^17^
		10	251.12	9.00 × 10^15^
		15	239.12	1.32 × 10^15^
	FWO	5	266.78	8.11 × 10^16^
		10	250.33	7.93 × 10^15^
		15	224.39	1.27 × 10^14^
	FRIEDMAN	5	281.24	8.99 × 10^17^
		10	247.75	5.07 × 10^15^
		15	231.84	4.15 × 10^14^
PP	KAS	5	177.03	1.22 × 10^11^
		10	179.54	1.50 × 10^11^
		15	178.29	1.44 × 10^11^
	FWO	5	178.25	2.84 × 10^12^
		10	183.11	1.55 × 10^12^
		15	180.27	2.07 × 10^11^
	FRIEDMAN	5	188.51	9.99 × 10^11^
		10	190.37	1.22 × 10^12^
		15	189.64	9.45 × 10^11^
PVC 1	KAS	5	89.24	2.23 × 10^7^
		10	106.95	1.62 × 10^9^
		15	107.99	2.19 × 10^9^
	FWO	5	89.31	2.27 × 10^7^
		10	107.03	1.65 × 10^9^
		15	107.99	2.20 × 10^9^
	FRIEDMAN	5	89.30	2.26 × 10^7^
		10	107.02	1.64 × 10^9^
		15	107.99	2.20 × 10^9^
PVC 2	KAS	5	190.08	5.07 × 10^12^
		10	140.07	1.44 × 10^9^
		15	127.29	2.06 × 10^8^
	FWO	5	158.12	1.83 × 10^10^
		10	140.08	1.44 × 10^9^
		15	127.41	2.10 × 10^8^
	FRIEDMAN	5	168.51	1.06 × 10^11^
		10	136.74	8.49 × 10^8^
		15	127.41	2.10 × 10^8^

**Table 4:** Values obtained from the thermodynamic parameters determined with the KAS model at different rates of heating of the different plastics.

Thermodynamic Parameters		Heating Rates K × min^−1^		
	(kJ × mol^−1^)	5	10	15
PS	H	166.252	196.439	194.105
	G	188.435	185.955	186.897
	S	−0.032	0.015	0.010
PET	H	223.307	224.409	204.847
	G	185.041	193.657	192.655
	S	0.055	0.044	0.017
HDPE	H	262.472	244.897	232.824
	G	214.105	211.552	211.255
	S	0.065	0.045	0.028
PP	H	171.169	173.562	172.279
	G	205.111	207.055	206.243
	S	−0.048	−0.217	−0.047
PVC	H	184.148	134.063	121.218
	G	210.114	209.467	209.313
	S	−0.036	−0.104	−0.121

## Data Availability

The data presented in this study are available on request from the corresponding author.
